# H63D Homozygous Mutation: An Unusual Cause of Deranged Liver Function Test in an Elderly Patient

**DOI:** 10.7759/cureus.31840

**Published:** 2022-11-23

**Authors:** Haris Nasrullah, Atif Nasrullah, Naeem Ijaz, Umar Hayat

**Affiliations:** 1 Hospital Medicine, University Hospitals Plymouth NHS Trust, Plymouth, GBR; 2 Internal Medicine, The Wright Center for Graduate Medical Education, Scranton, USA; 3 Gastroenterology, Geisinger Medical Center, Wilkes-Barre, USA

**Keywords:** elevated lft, phlebotomy in elderly, elderly patient, iron overload, hemochromatosis, h63 homozygous mutation

## Abstract

Hereditary hemochromatosis is an autosomal recessive disorder characterized by dysregulated iron homeostasis resulting in body iron overload. Hemochromatosis leads to excessive iron deposition in the parenchymal cells of different body organs, resulting in the compromise of their normal functioning in genetically predisposed patients. It presents in genetically predisposed male patients aged between 40 and 70 years. Various mutations have been described in hemochromatosis, *C282Y* is the most prevalent and is commonly associated with iron overload. Other mutations such as *H63D* and *S65C* rarely lead to iron overload in patients. We present an unusual case of an 84-year-old male who was referred for comprehensive evaluation. He was found to have mildly elevated liver function tests (LFTs). Further workup revealed raised ferritin levels, and on a detailed investigation, it was found to be homozygous for the *H63D *mutation for hemochromatosis. The patient was seen by hematology and was treated with therapeutic phlebotomy, which led to the normalization of the LFTs and improvement in ferritin levels and clinical symptoms.

## Introduction

Hereditary hemochromatosis is a well-defined autosomal recessive disorder characterized by dysregulated iron homeostasis resulting in body iron overload [1].^ ^It can cause excessive iron deposition in the parenchymal cells of different body organs, compromising their normal functioning. The most commonly affected body organs are the liver, heart, pancreas, and other endocrine organs. Iron overload in cells leads to a cascade of events, starting from the formation of toxic metabolites resulting in cell death [2,3]. This cascade continues to manifest as diverse tissue pathologies and clinical syndromes such as diabetes mellitus, cirrhosis, congestive heart failure, erectile dysfunction, hepatocellular carcinoma (HCC), chondrocalcinosis, etc. 

The prevalence of hereditary hemochromatosis is about 1 per 300 persons in the USA, Europe, and Australia [4,5]. von Recklinghausen used the term *hemochromatosis* for widespread tissue injury due to iron overload [6]. Feder et al. identified the first mutation in hemochromatosis in 1996 [7]. Since then, multiple mutations in hemochromatosis have been identified, with the *C282Y* gene mutation being the most common (80%-85%) and is mainly associated with iron overload (10%). Approximately 70% of the *C282Y* homozygotes can phenotypically express the disease [8]. In contrast, mutations in *H63D* and *S65C* genes are present in fewer patients [9,10].

The clinical presentation of hemochromatosis is variable and depends on the organ involved; however, most patients present with nonspecific initial symptoms such as fatigue and arthralgia. The most common first organ involved in hemochromatosis is the liver. Liver function test (LFT) derangement leads to diagnostic workup in most patients. The mainstay of treatment for phenotypic disease expression is therapeutic phlebotomy. Here, we present a case of an 84-year-old male with a deranged LFT. The patient had new diabetes the last one year, well controlled with a minimal dose of metformin. The patient was further worked up for deranged liver functions and found to have raised ferritin levels. On extensive investigation, the patient was found to be positive for *H63D *homozygous genetic mutation. The patient was successfully treated with phlebotomy, which improved symptoms and laboratory parameters.

## Case presentation

An 84-year-old male with a past medical history of diabetes mellitus type 2, paroxysmal atrial fibrillation, Wolff Parkinson white syndrome with past ablation, dilated cardiomyopathy with dual-chamber pacemaker insertion (last echocardiogram [ECHO] ejection fraction [EF] 50%), hypertension, hyperlipidemia, benign prostatic hypertrophy, colon cancer with the previous hemicolectomy, and osteoarthritis (multiple joints). He presented to our clinic for a comprehensive geriatric evaluation. The patient reported that he had been feeling fatigued and lethargic for the past few months. The patient denied any recent skin pigmentation, visual problems, or sexual dysfunction. The patient was on multiple medications for chronic medical conditions, including warfarin for atrial fibrillation, escitalopram for depression, atorvastatin for hyperlipidemia, and metformin 500 mg daily for type 2 diabetes. The patient stated that he was diagnosed with diabetes mellitus about one year ago, with a maximum HbA1c reading of 7.3, and his blood sugar was well controlled with metformin. The in-clinic evaluation showed stable vitals, osteoarthritic changes in the hands, and irregularly irregular heart rate with no evidence of skin pigmentation, signs of joint inflammation, or portal hypertension. The patient had ambulatory dysfunction due to osteoarthritis, bilateral hip replacement, and previous spinal surgery, but no evidence of peripheral neuropathy was found on examination. The patient denied any recent history of alcohol intake and herbal or over-the-counter medication use. The patient had stable chronic kidney disease (CKD) stage 3 for a long time with no recent change in kidney function. The initial laboratory workup showed deranged LFTs, and repeat LFT tests showed a similar picture (Table 2). A review of previous medical records showed intermittent derangement in LFTs for the last few years, and USG showed normal portal vein size and no evidence of cirrhosis/ascites. On detailed workup, the patient had elevated ferritin levels and tested positive for hemochromatosis (*H63D* homozygous mutation) on genetic testing (Table 4) and negative for *C282Y* and *S65C*. The evaluation showed negative screening for hepatitis B and C, normal Alpha 1 antitrypsin levels, and ceruloplasmin levels. The patient was referred to a hematologist for further evaluation and, as per the hematologist's recommendations, was treated with phlebotomy, which improved LFTs, ferritin levels, and clinical symptoms.

**Table 1:** Complete blood count. WBC, white blood cell

Test	Value
Hemoglobin	14.1 g/dL
Hematocrit	43.5
WBC count	5.9 × 10^3^ microL
Platelet count	143 × 10^3^ microL

**Table 2:** Liver function test. AST, aspartate transaminase; ALT, alanine transaminase; u, units; INR, international normalized ratio

Liver function test	Day 1 (Initial test)	Day 60 (Repeat test)	After phlebotomy
ALT	94 u/L	67 u/L	24 u/L
AST	89 u/L	56 u/L	28 u/L
Albumin	4.4 g/dL	3.8 g/dL	3.7 g/dL
Alkaline phosphatase	65 u/L	72 u/L	46 u/L
Bilirubin	0.7 mg/dL	1 mg/dL	0.68 mg/dL
INR	2.27		

**Table 3:** Iron studies. TIBC, total iron binding capacity

	On initial evaluation	Repeat tests before treatment	After phlebotomy
Ferritin (normal range 30-400)	1,707 ng/mL	1,668 ng/mL	424 ng/mL
Transferrin saturation (normal range 15%-50%)	49%	46%	37%
TIBC (normal range 250-425 microg/dL)	285 microg/dL	275 microg/dL	166 microg/dL
Iron (normal range 45-176 microg/dL)	139 microg/dL	127 microg/dL	Not available

**Table 4:** Mutation testing.

Hemochromatosis PCR	
Hemochromatosis mutation C282Y	Negative
Hemochromatosis mutation H63D	Homozygous positive
Hemochromatosis mutation S65C	Negative

**Table 5:** Other workup. ELISA, enzyme-linked immunosorbent assay

Test	Value
Hepatitis C ELISA	Nonreactive
Hepatitis B ELISA	Nonreactive
Ceruloplasmin	19.4 mg/dL (range 16-31)
Alpha 1 antitrypsin levels	127 mg/dL (range 101-187)

## Discussion

Hepcidin is a peptide synthesized in the liver and is involved in mediating a complex interplay of the cellular pathway of iron homeostasis. The synthesis of hepcidin itself is also regulated by serum iron and stored iron levels in the body through a complex feedback mechanism. When hepcidin is bound to a transmembrane iron transport receptor (ferroportin), it activates the feedback signaling pathway, resulting in the degradation of ferroportin, thus preventing excessive iron absorption in the body. The genetic mutation in the hereditary hemochromatosis involving the hepcidin gene can cause dysregulation of this iron homeostasis, resulting in excessive iron absorption. It leads to excessive body iron and ferritin, leading to iron deposition in the body organs and causing organ dysfunction [11].

In 1865, Trousseau mentioned the association between liver disease, skin changes, and diabetes mellitus [12]. In 1889, the term *hemochromatosis* was used for the first time by von Recklinghausen for widespread tissue injury caused by iron overload in different body organs of patients [6]. Furthermore, in 1996, Feder et al. identified the gene responsible for hemochromatosis [7].

Hereditary hemochromatosis is divided into five major categories: type 1, being the most common and is related to the homeostatic iron regulator (HFE); type 2 is called Juvenile and is present in young patients; type 3 is transferrin receptor-related; type 4, ferroprotein disease; and type 5, aceruloplasminemia due to a heterozygous mutation in the *FTH1* gene on chromosome 11q12 [13].^ ^Over the years, multiple gene mutations have been identified as a culprit causing hemochromatosis, with *C282Y *most commonly present in patients and associated with iron overload-induced organ dysfunction. Other common mutations identified are *H63D* and *S65C*, but these are not commonly associated with iron overload. *H63D* and *S65C* mutations can present separately or in heterozygote forms with *C282Y* mutations. Homozygous forms of *H63D* and *S65C* are rarely associated with iron overload and organ damage [13-15].

In a study reported by Kelley et al., 4,138 individuals underwent the HFE genotype, and they found 366 (8.8%) individuals to have homozygous forms for the *C282Y* gene mutation and 170 (4.1%) positive for the *H63D* homozygous genetic mutation. Among *H63D* homozygous mutated patients, only three had evidence of iron overload and only one met the criteria for iron overload-related disease organ damage [16].

While working on patients with raised ferritin levels, Gochee et al. found that male patients with *H63D* homozygous mutation had elevated transferrin saturation compared to other patients [14]. The iron overload study (Hemochromatosis and Iron Overload Screening [HEIRS]) found ferritin and transferrin saturation to be higher in *H63D* compound heterozygote and homozygote patients than in hemochromatosis patients with wild-type mutation [17].^ ^A recent meta-analysis by Diamandis et al. emphasized that H63D mutation leads to widespread tissue injury [17]. Powell et al. reported that *H63D *mutation is associated with cognitive impairment, cardiac abnormalities, and sexual problems. They also reported that *H63D* mutation should not be ignored and that patients should be effectively worked up and treated [18].

Additionally, Aguilar-Martinez et al. demonstrated that *H63D* homozygous mutation was associated with a wide range of iron overload. The study showed that 24% of the patients were *H63D* positive, higher than previously reported studies. However, the patients of this study were found to have raised iron levels at the baseline and were referred for workup [19]. Secondary causes of iron overload include multiple transfusions, and various forms of anemias: thalassemia, congenital sideroblastic anemia, myelodysplastic syndromes, aplastic anemias, etc.

Our case is unique because it had end-organ damage with deranged LFTs, diabetes, dilated cardiomyopathy, and atrial fibrillation. The patient was referred to hematology and had therapeutic phlebotomy, which led to improved symptoms, LFTs, and serum ferritin levels.

## Conclusions

Hereditary hemochromatosis is a well-defined autosomal recessive disorder with variable penetrance. We emphasize that elderly patients with abnormal LFTs and new-onset diabetes mellitus should be worked up for and looked up for unusual causes like hemochromatosis. Early diagnosis can improve the quality of care and save healthcare resources.
